# An Infrared-Visible Image Registration Method Based on the Constrained Point Feature

**DOI:** 10.3390/s21041188

**Published:** 2021-02-08

**Authors:** Qingqing Li, Guangliang Han, Peixun Liu, Hang Yang, Huiyuan Luo, Jiajia Wu

**Affiliations:** 1 Changchun Institute of Optics, Fine Mechanics and Physics, Chinese Academy of Sciences, Changchun 130033, China; liqingqing17@mails.ucas.ac.cn (Q.L.); liupx@ciomp.ac.cn (P.L.); yanghang@ciomp.ac.cn (H.Y.); luohuiyuan@ciomp.ac.cn (H.L.); wujiajia17@mails.ucas.ac.cn (J.W.); 2 School of Optoelectronics, University of Chinese Academy of Sciences, Beijing 100049, China

**Keywords:** infrared-visible registration, object detection, constrained points, LV-rule, evaluation method

## Abstract

It is difficult to find correct correspondences for infrared and visible image registration because of different imaging principles. Traditional registration methods based on the point feature require designing the complicated feature descriptor and eliminate mismatched points, which results in unsatisfactory precision and much calculation time. To tackle these problems, this paper presents an artful method based on constrained point features to align infrared and visible images. The proposed method principally contains three steps. First, constrained point features are extracted by employing an object detection algorithm, which avoids constructing the complex feature descriptor and introduces the senior semantic information to improve the registration accuracy. Then, the left value rule (LV-rule) is designed to match constrained points strictly without the deletion of mismatched and redundant points. Finally, the affine transformation matrix is calculated according to matched point pairs. Moreover, this paper presents an evaluation method to automatically estimate registration accuracy. The proposed method is tested on a public dataset. Among all tested infrared-visible image pairs, registration results demonstrate that the proposed framework outperforms five state-of-the-art registration algorithms in terms of accuracy, speed, and robustness.

## 1. Introduction

Multi-modal image fusion can offer a composited and detailed scene representation to improve the accuracy of decision-making in subsequent tasks [1]. This technique is widely applied in the fields of medical diagnosis [2,3], remote sensing image processing [4,5], and surveillance [6,7]. Image registration is an essential step to ensure fusion operation, which aligns two or more images from different times, sensors, and views by finding a credible spatial transformation [8]. However, due to the complementary information and different imaging principles of multi-sensor images, the mutual information of infrared and visible images is less [9]. It is a challenge to find correspondences for infrared-visible image registration [10]. Therefore, this paper aims to design a framework for realizing high-precision infrared-visible registration.

Numerous methods have been proposed to improve the accuracy, speed, and robust performance of image registration. These methods can be generally divided into intensity-based, deep learning-based, and feature-based methods [11].

The intensity-based methods register images by constructing a similarity measurement function. Normalized cross-correlation (NCC) [12], phase correlation (PC) [13,14], and mutual information (MI) [15,16] are three classical functions used in intensity-based methods. However, methods based on these conventional functions cannot deal with obvious structure inconsistency in multispectral or multi-modal images. To handle the problem, Chen et al. [17] proposed a normalized total gradient (NTG) function that obtains good results in the multi-spectral image registration and spectral color measurement [18]. Despite its advantage, computing costs are increased. In general, these intensity-based methods are sensitive to image distortion, appearance change caused by noise, varying illumination, and different imaging sensors. The pixel information varies greatly between infrared and visible images. Thus, intensity-based methods are not suitable for infrared-visible image registration.

Many deep learning network models have been proposed for image registration in recent years. Fan et al. [19] designed a BIRNet to align two brain images, which employs a novel hierarchical dual-supervised fully convolutional neural network (FCN) [20] to train data and a basic U-Net [21] model to improve accuracy and efficiency. Wang et al. [22] developed a deep learning method to register remote sensing images by directly learning the mapping between patch-pairs and labels. In general, these deep learning-based methods have served for medical and remote sensing image registration [23,24]. However, the optical characteristics, geometric characteristics, and spatial location expressed by infrared and visible images are very different [25]. Methods based on deep learning networks are limited when applied to wide baseline image registration and it is difficult to get the spatial relationships between two or more points with convolutional neural networks (CNNs). Therefore, deep learning methods face many challenges of achieving excellent infrared-visible image registration [11].

Feature-based methods are very popular owing to their strong robustness, flexibility, and the ability of wide applications. These methods determine spatial transformation parameters according to the correspondence features. Point features [26], edge features [27], and morphological region features [28] are three dominant features. Since points are easier to extract and describe with a simplified form than the other two features, the point feature becomes the commonly used feature [11]. The Harris corner is a representative point feature due to its illumination insensitivity and rotation invariance. The corner feature is applied in remote sensing image registration [29,30]. Pei et al. [31] improved the Harris corner to solve the corner clustering problem and accelerate the registration speed. Scale invariant feature transform (SIFT) is another representative point feature for its robustness to the change of scale, illumination, and rotation [32,33]. Lv et al. [34] developed a strategy by combining the gradient information and the SIFT feature to improve the registration accuracy. With the development of computer technology and the increasing requirement of image registration tasks, some modified SIFT descriptors have been presented continuously. The speed up robust feature (SURF) based on SIFT is proposed to reduce the computation and align the color images [35,36]. Ke et al. [37] designed a PCA-SIFT descriptor that unites a principal component analysis algorithm and SIFT to decrease dimensions and memory footprints of feature descriptors and increase the matching speed. To acquire accurately matched points for remote sensing images, Ma et al. [38] presented an enhanced feature-matching method named PSO-SIFT. However, the extraction speed of the abovementioned features cannot satisfy real-time video stream processing. Rosten and Drummond [39] provided a feature descriptor called “features from accelerated segment test” (FAST) to obviously speed up the speed of feature detection. Rublee et al. [40] presented the ORB algorithm, which introduces the orientation to the FAST method to enhance the robustness of the environmental variation. As mentioned above, various kinds of point features are proposed for image registration. Traditional point feature registration methods guarantee accuracy by constructing complex feature descriptors or accelerating the speed by simplifying the descriptors. Thus, it is worth researching how to balance accuracy and speed of image registration.

How to qualitatively and quantitatively evaluate the registration algorithms is also significant. Torabi et al. [41] presented a simple and accurate evaluation strategy by calculating the overlap ratio of binary polygons. The method is used to estimate registration accuracy of infrared-visible images [42]. The drawback of this method is that it needs to manually select matched points to construct polygons, which takes plenty of time and energy. 

The information of infrared and visible images is often quite diverse because of their imaging principles. Therefore, many methods have difficulty achieving satisfactory registration results of infrared-visible images. This paper proposes a framework based on the constrained point feature for high precision and speed registration of infrared-visible images. Constrained points are first captured by utilizing an object detection algorithm. Then the LV-rule is presented to match constrained points. Finally, the transformation matrix is determined depending on the matched points, which is used to align the infrared and visible images. Furthermore, we put forward an intelligent method based on reference [41] to automatically evaluate the registration accuracy.

Our contributions in this paper are summarized as follows:

1. An infrared-visible image registration framework based on the constrained point feature is proposed. Constrained points are obtained by adopting an object detection algorithm to avoid designing the complex feature descriptor and introduce the senior semantic information to improve the registration accuracy.
2. The LV-rule is designed to match constrained points strictly without eliminating the mismatched and redundant points, which increases the registration speed.
3. An automatic method is presented to evaluate the accuracy of infrared-visible image registration.

The rest of this article is structured as follows: Section 2 analyzes the practicability of the proposed method, gives approaches to extract and match constrained points, and describes the evaluation criteria. Section 3 provides the experimental results and analysis, including the feature points extraction and matching experiment and the image registration experiment. Section 4 discusses the research work and the results of the experiments. Section 5 presents the conclusions of our method.

## 2. Methodology

This paper proposes a method based on the constrained point feature to carry out accurate and fast infrared-visible image registration. The definition of the constrained point feature is explained in detail as follows. The detection result of each object is a bounding box with four corner points. The coordinates of the four points are constrained by the location information of the object. Therefore, the corner of the bounding box is defined as the constrained point feature, which also can be called the constrained point.

### 2.1. The Workflow of the Proposed Method

**Figure 1:**
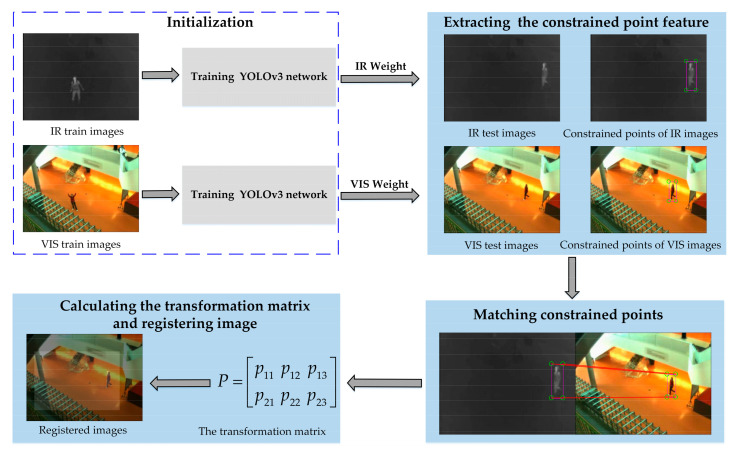
The flowchart of the proposed method. (IR means the infrared image and VIS means the visible image in this article).

The proposed method aligns the infrared and visible images base on constrained points, which are captured by utilizing the object detection approach. As shown in Figure 1, the proposed framework mainly consists of three parts.

1. Extracting the constrained point feature: Instead of designing the complex feature descriptor, the constrained point feature is extracted from the object bounding box obtained by the object detection method. Bounding boxes contain the location information which is considered the senior semantic information to increase the registration accuracy. We employ a high-precision and fast object detection method named YOLOv3 [43,44] to acquire constrained points. To obtain accurate detection results, the YOLOv3 model is first retrained. Detailed information about retraining YOLOv3 model is introduced in Section 2.3. Then, the testing images are sent to the retrained YOLOv3 model to get object bounding boxes.
2. Matching constrained points: The LV-rule is constructed to match constrained points, which avoids the elimination operation of mismatched and redundant point pairs to decrease the matching time.
3. Calculating the transformation matrix and registering image: An affine transformation model is used to get the spatial transformation of infrared and visible images. The affine transformation matrix P is calculated depending on the matched point pairs. The matrix is used to obtain the aligned image.

### 2.2. The Practicability Analysis of the Proposed Method

For the image registration task, the spatial mapping relationship between two images can be expressed as follows:

(1) g(w,z)=Trans(f(x,y))

where f(x,y) and g(w,z) represent the float (infrared) and reference (visible) images, respectively. (x,y) and (w,z) refer to the coordinates corresponding to the pixels of the two modal images, respectively. Trans() is the transformation model. Therefore, the image registration task can be described as a problem for solving the transformation model Trans().

Affine transformation [9] is commonly used because it can maintain the fixed linear state and parallel relation in the image before and after transformation. The affine transformation model includes four types of image transformations: translation, rotation, scaling, and shearing. The affine transformation model is shown in [18]

(2) $$\Phi_{R}=P*\Phi_{F}$$

The explicit expression of Equation (2) is

(3) $$\left(\begin{array}{c} w_{1}\;w_{2}\;w_{3}\;w_{4}\;\cdots \;w_{i}\;\cdots \;w_{n}\;\\ z_{1}\;\;{\tilde{z}}_{2}\;\;{\tilde{z}}_{3}\;\;z_{4}\;\;\cdots \;z_{i}\;\cdots \;z_{n} \end{array}\right)=\left(\begin{array}{c} p_{11}\;p_{12}\;p_{13}\\ p_{21}\;p_{22}\;p_{23} \end{array}\right)*\left(\begin{array}{c} x_{1}\;x_{2}\;x_{3}\;x_{4}\;\cdots \;x_{i}\;\cdots \;x_{n}\\ y_{1}\;y_{2}\;y_{3}\;y_{4}\;\cdots \;y_{i}\;\cdots \;y_{n}\\ 1\;\;\;1\;\;\;1\;\;\;1\;\;\cdots \;1\;\;\cdots \;1 \end{array}\right)i=1,2,3,\cdots n$$

where P is the affine transformation matrix, $\Phi_{R}$ is the feature points set of the reference image, $\Phi_{F}$ is the feature points set of the float image, and *n* is the number of points. 

There are 6 parameters of the affine transformation matrix P. Thus, if the affine transformation matrix P has a unique solution, the rank of the matrix $\Phi_{F}$ should be 3. That is,

(4) $$rank(\Phi_{F})=3$$

In other words, at least three feature points are not on the same line. Obviously, the proposed registration method satisfies the above condition because each object bounding box contains four corners, three of which are not available on an identical straight line. This verifies the theoretical feasibility of the proposed framework in this paper.

For a pair of correctly matched points $\left(x_{i},y_{i}\right)$ and $\left(w_{i},z_{i}\right)$, Equation (3) can be described as

(5) $$\left\{\begin{array}{c} w_{i}=\;p_{11}x_{i}+p_{12}y_{i}+p_{13}\\ z_{i}=\;\;p_{21}x_{i}+p_{22}y_{i}+p_{23} \end{array}\right.$$

Therefore, it is not difficult to solve the parameters of the matrix P. The key step to carrying out high precision registration of infrared-visible images is obtaining valid and accurately matched feature point pairs.

### 2.3. Extracting the Constrained Points 

This paper presents an artful method to extract the constrained point feature by utilizing the object algorithm. Object detection methods can be used to obtain bounding boxes and capture constrained points without requesting intricate feature descriptors. The position information contained in the object bounding box is considered high-level semantic information, which guarantees the precision of image registration. Therefore, in the proposed framework, an accurate detection method that can obtain exact object bounding boxes is required. YOLOv3 is a very strong detector that excels at producing decent boxes for objects. Meanwhile, YOLOv3 has high detection accuracy and speed [44,45]. Thus, this study adopted the YOLOv3 model to obtain the object bounding boxes.

**Figure 2:**
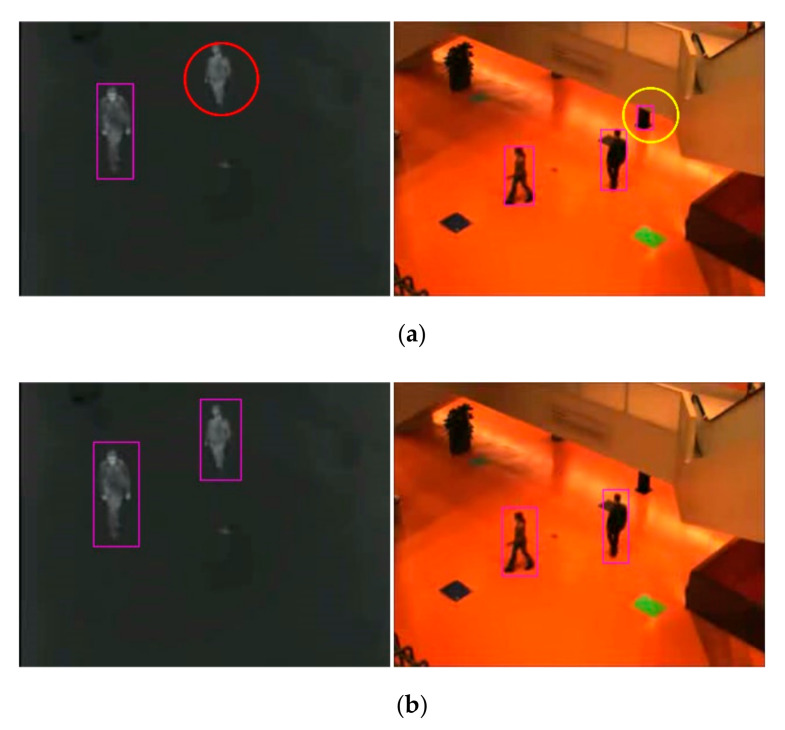
Object detection results: (**a**) detection results of the pre-trained model and (**b**) detection results of the retrained model.

The pre-trained model of YOLOv3 was first used to detect objects for infrared and visible images. The pre-trained YOLOv3 model was trained on the COCO dataset. Figure 2 shows the comparison of the detection results between the pre-trained YOLOv3 model and the retrained YOLOv3 model, where the carmine box is the object detection result, the red circle is the missing object, and the yellow circle is the false object. It can be seen from Figure 2 that the pre-trained YOLOv3 model resulted in missing and false objects. In order to get precise detection results and accurately capture the constraint points, the YOLOv3 model was retrained. 

The YOLOv3 model was retrained depending on a GPU (NVIDIA GeForce GTX 1070). We trained the YOLOv3 model on the LITIV dataset, a public dataset for infrared-visible image registration and object tracking [41]. The size of the images was 320 × 240. A total of 1200 infrared images were selected as training samples to get the IR weight model. A total of 1200 visible images were selected as training samples to get the VIS weight model. Training samples were marked using the label tool provided by [44]. At the training stage, the batch size was set to 16. 

YOLOv3, SSD, and Faster R-CNN are three dominant deep learning object detection algorithms [46,47]. We compared the detection performance of YOLOv3 to SSD and Faster R-CNN. Mean accuracy (MA) was used to evaluate the detection accuracy, which is expressed as follows:

(6) $$MA=\frac{\sum_{i=1}^{NI}A(i)}{NI}\times 100\%\;\;\;\;\;\;\;\;\;\;\;\;i=1,2,3,\cdots ,NI$$

(7) $$A=\frac{the\;number\;of\;objects\;detected\;correctly\;in\;an\;image\;}{the\;number\;of\;objects\;in\;an\;image}$$

where *NI* is the number of testing images.

A total of 300 infrared and 300 visible images of the LITIV dataset were used to test detection accuracy and speed. Table 1 provides the comparison of the MA and time values for SSD, Faster R-CNN, and YOLOv3. The time value expresses the average running time to detect an image. As can be seen from Table 1, for infrared images, the MA value of YOLOv3 was equal to Faster R-CNN and improved 0.72% more than SSD; for visible images, the MA value of YOLOv3 increased 1.34% and 0.5% more than SSD and Faster R-CNN, respectively. The time value of YOLOv3 was much less than SSD and Faster R-CNN. In summary, these three methods all had fine performance to detect objects, but YOLOv3 had higher detection accuracy and faster detection speed than SSD and Faster R-CNN. As a result, this paper adopted the YOLOv3 network to obtain object bounding boxes.

**Figure 3:**
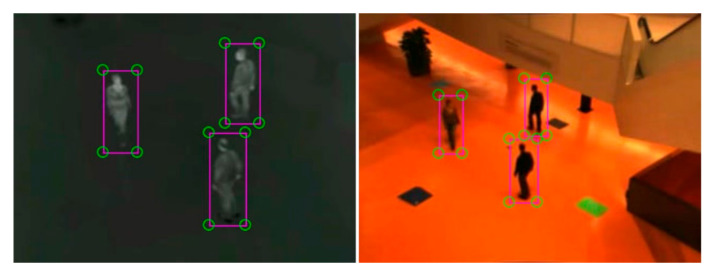
The constrained points extraction results of infrared (**Left**) and visible (**Right**) images. (The carmine-colored boxes are object bounding boxes, and the green circles are constrained points).

While extracting constrained points, the retrained IR and VIS YOLOv3 models were separately sent to the YOLOv3 network to detect objects in infrared and visible images. The four corners of each object bounding box were defined as the constrained points. Figure 3 exhibits the extraction results of constraint points from a pair of infrared and visible images. The YOLOv3 network provided accurate position information of objects, which ensured the registration accuracy. 

### 2.4. Matching the Constrained Points

This paper provides an LV-rule method for matching constrained points, which avoids eliminating the mismatched and superfluous points and improves matching accuracy and speed. Next, we specifically introduce the LV-rule matching method.

**Figure 4:**
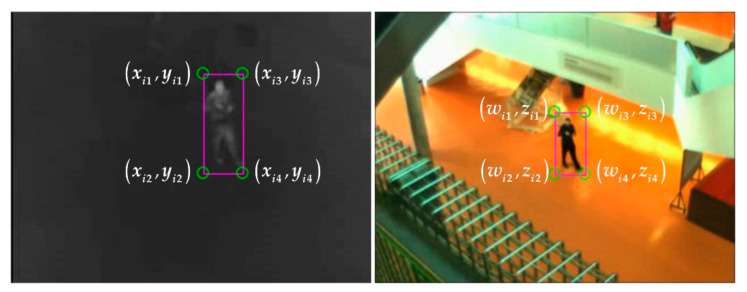
The coordinate description of the object bounding boxes in infrared (**Left**) and visible (**Right**) images.

As shown in Figure 4, $x_{i1}=x_{i2}$,$w_{i1}=w_{i2}$,$\left(x_{i1},y_{i1}\right)$, and $\left(w_{i1},z_{i1}\right)$ are the left-top corner points of the object bounding boxes in the infrared and visible images, respectively. $x_{i1}$ and $w_{i1}$ are separately defined as the left values of infrared and visible images. 

The core idea of matching constrained points is that the object bounding box with the minimum left value of the visible image corresponds to the object bounding box with the minimum left value of the infrared image. Therefore, all left values of an image are sorted to get the order of bounding boxes. The object bounding boxes are matched one by one from the order. Since the corner position relationship of each object bounding box is fixed, the constrained points are matched according to the matched object bounding box pairs. The above registration idea is defined as the LV-rule. The mathematical model of the LV-rule matching method is described as follows.

The set of the object bounding boxes from an image is represented as

(8) $$\Phi^{2\times N}=\left(\phi_{1},\phi_{2},\phi_{3},\cdots \phi_{i},\cdots \phi_{n}\right)\;\;\;\;\;\;\;\;\;\;i=1,2,3,\cdots ,n$$

(9) $$\phi_{i}=\left(x_{i1}\;\;y_{i1};x_{i2}\;\;y_{i2};x_{i3}\;\;y_{i3};x_{i4}\;\;y_{i4}\right)$$

where $\phi_{i}$ refers to the coordinate information of an object bounding box, n is the number of the object bounding boxes of an image, and N=4n is the number of constrained points of an image. Thus, $\Phi^{2\times N}$ is a 2×N matrix.

The set of all left values from one image is written as

(10) $$\Psi =\left(x_{11},x_{21},x_{31},\cdots ,x_{i1},\cdots ,x_{n1}\right)\;\;\;\;\;\;\;i=1,2,3,\cdots ,n$$

The matrix of sorted left values $\tilde{\Psi }$ is given by

(11) $$\tilde{\Psi }=S(\Psi )$$

in which S is a function to sort the left values.

The sets of sorted left values and matched object bounding boxes from infrared and visible images are denoted as

(12) $$\left\{\begin{array}{c} {\tilde{\Psi }}_{IR}\;=\left({\tilde{x}}_{11},{\tilde{x}}_{21},{\tilde{x}}_{31},\cdots ,{\tilde{x}}_{i1},\cdots ,{\tilde{x}}_{n1}\right)\\ {\tilde{\Psi }}_{VIS}=\left({\tilde{w}}_{11},{\tilde{w}}_{21},{\tilde{w}}_{31},\cdots ,{\tilde{w}}_{i1},\ldots ,{\tilde{w}}_{n1}\right) \end{array}\right.\;\;\;\;\;\;\;\;\;\;\;\;i=1,2,3,\;\cdots ,n$$

(13) $$\left\{\begin{array}{c} {\tilde{\Phi }}_{IR}^{2\times N}=\left({\tilde{\phi }}_{1},{\tilde{\phi }}_{2},{\tilde{\phi }}_{3},\cdots {\tilde{\phi }}_{i},\cdots {\tilde{\phi }}_{n}\right)\\ {\tilde{\Phi }}_{VIS}^{2\times N}=\left({\tilde{\varphi }}_{1},{\tilde{\varphi }}_{2}\;,{\tilde{\varphi }}_{3},\cdots {\tilde{\varphi }}_{i},\cdots {\tilde{\varphi }}_{n}\right) \end{array}\right.\;\;\;\;\;\;\;\;\;\;\;\;\;\;\;i=1,2,3,\;\cdots ,n$$

where ${\tilde{x}}_{i1}$ matches with ${\tilde{w}}_{i1}$, ${\tilde{x}}_{11}\leq {\tilde{x}}_{21}\leq {\tilde{x}}_{31}\cdots \leq {\tilde{x}}_{i1}\cdots \leq {\tilde{x}}_{n1}$, ${\tilde{\phi }}_{i}$ matches with ${\tilde{\varphi }}_{i}$, ${\tilde{\phi }}_{i}$ is a bounding box corresponding with left value ${\tilde{x}}_{i1}$ in the infrared image, and ${\tilde{\varphi }}_{i}$ is a bounding box corresponding with the left value ${\tilde{w}}_{i1}$ in the visible image.

According to Formulas (9) and (13), the matched constrained points of infrared and visible images can be expressed as

(14) $$\left\{\begin{array}{c} \begin{array}{c} {\tilde{\Phi }}_{VIS}^{2\times N}=\left(\begin{array}{c} {\tilde{x}}_{11}\;{\tilde{x}}_{12}\;{\tilde{x}}_{13}\;{\tilde{x}}_{14}\;{\tilde{x}}_{21}\;{\tilde{x}}_{22}\;{\tilde{x}}_{23}\;{\tilde{x}}_{24}\;\cdots \;{\tilde{x}}_{i1}\;{\tilde{x}}_{i2}\;{\tilde{x}}_{i3}\;{\tilde{x}}_{i4}\;\cdots \;{\tilde{x}}_{n1}\;{\tilde{x}}_{n2}\;{\tilde{x}}_{n3}\;{\tilde{x}}_{n4}\;\\ {\tilde{y}}_{11}\;{\tilde{y}}_{12}\;{\tilde{y}}_{13}\;{\tilde{y}}_{14}\;{\tilde{y}}_{21}\;{\tilde{y}}_{22}\;{\tilde{y}}_{23}\;{\tilde{y}}_{24}\;\cdots \;{\tilde{y}}_{i1}\;{\tilde{y}}_{i2}\;{\tilde{y}}_{i3}\;{\tilde{y}}_{i4}\;\cdots \;{\tilde{y}}_{n1}\;{\tilde{y}}_{n2}\;{\tilde{y}}_{n3}\;{\tilde{y}}_{n4\;} \end{array}\right)\\ {\tilde{\Phi }}_{VIS}^{2\times N}=\left(\begin{array}{c} {\tilde{w}}_{11}\;{\tilde{w}}_{12}\;{\tilde{w}}_{13}\;{\tilde{w}}_{14}\;{\tilde{w}}_{21}\;{\tilde{w}}_{22}\;{\tilde{w}}_{23}\;{\tilde{w}}_{24}\;\cdots \;{\tilde{w}}_{i1}\;{\tilde{w}}_{i2}\;{\tilde{w}}_{i3}\;{\tilde{w}}_{i4}\;\cdots \;{\tilde{w}}_{n1}\;{\tilde{w}}_{n2}\;{\tilde{w}}_{n3}\;{\tilde{w}}_{n4}\;\\ {\tilde{z}}_{11}\;{\tilde{z}}_{12}\;{\tilde{z}}_{13}\;{\tilde{z}}_{14}\;{\tilde{z}}_{21}\;{\tilde{z}}_{22}\;{\tilde{z}}_{23}\;{\tilde{z}}_{24}\;\cdots \;{\tilde{z}}_{i1}\;{\tilde{z}}_{i2}\;{\tilde{z}}_{i3}\;{\tilde{z}}_{i4}\;\cdots \;{\tilde{z}}_{n1}\;{\tilde{z}}_{n2}\;{\tilde{z}}_{n3}\;{\tilde{z}}_{n4\;} \end{array}\right) \end{array} \end{array}\right.i=1,2,3,\cdots ,n$$

### 2.5. Evaluation Criteria

In this paper, a registration error (RE) is defined to qualitatively evaluate the accuracy of image registration, and the root mean squared error (RMSE) is used as a reference to demonstrate the effectiveness of the new evaluation method. In addition, an effective registration rate (ERR) is defined to qualitatively evaluate the robustness of the proposed framework.

#### 2.5.1. Registration Error

**Figure 5:**
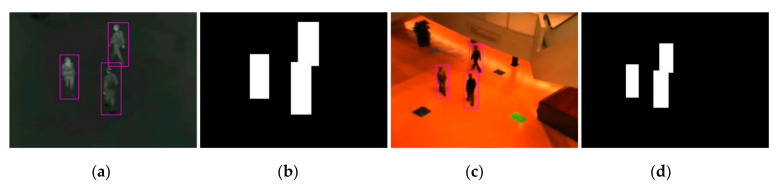
Example of evaluation polygons obtained by proposed evaluation method: (**a**) object detection results of the infrared image, (**b**) the threshold polygons image of the infrared image, (**c**) object detection results of the visible image, and (**d**) the threshold polygon image of the visible image.

This paper establishes a new evaluation system based on the literature [41]. The bounding boxes obtained by the object detection algorithm are used to get the threshold polygon for evaluating the registration accuracy. Compared with the reference [41], the advantage of our evaluation method is that the evaluation polygon is not required to be marked manually, and the final registration error value can be obtained automatically. The threshold polygons of one pair of infrared and visible images are shown in Figure 5. 

An overlap ratio parameter OR is defined to represent the overlap degree of the evaluation rectangle corresponding to the registered and reference images.

(15) $$OR=\frac{\sum_{i=1}^{a}\sum_{j=1}^{b}\Lambda \left(r_{i,j},v_{i,j}\right)}{\sum_{i=1}^{a}\sum_{j=1}^{b}ϒ\left(r_{i,j},v_{i,j}\right)}$$

(16) $$\Lambda \left(r_{i,j},v_{i,j}\right)=\left\{\begin{array}{c} 1,\;\;\;\;\;(r_{i,j}=1,v_{i,j}=1)\\ 0,\;\;\;\;\;others \end{array}\right.$$

(17) $$ϒ\left(r_{i,j},v_{i,j}\right)=\left\{\begin{array}{c} 0,\;\;\;\;\;(r_{i,j}=0,v_{i,j}=0)\\ 1,\;\;\;\;\;others \end{array}\right.$$

where $r_{i,j}$ is the pixel value at the coordinate (i,j) in the threshold polygon image corresponding to the registered infrared image, $v_{i,j}$ is the pixel value at the coordinate (i,j) in the threshold polygon image corresponding to the visible image, and a×b is the size of the test image.

The relationship between the registration error RE and the overlap ratio OR is expressed as

(18) RE=1−OR

#### 2.5.2. Root Mean Squared Error

The root mean squared error (RMSE) [48] is used to evaluate the accuracy of the proposed registration framework and verify the availability of the presented evaluation method.

(19) $$RMSE=\sqrt{\frac{1}{N}\sum_{i=1}^{N}{\left(x_{i}^{\prime }-x_{i}\right)}^{2}+{\left(y_{i}^{\prime }-y_{i}\right)}^{2}}$$

where *N* is the number of verification points, $\left(x_{i},y_{i}\right)$ are the coordinates of the validation points in the reference image, and $\left(x_{i}^{\prime },y_{i}^{\prime }\right)$ are the coordinates of the corresponding points in the registered image. The constrained point is applied to the validation points in this paper.

#### 2.5.3. Effective Registration Rate 

The effective registration rate (ERR) is given to evaluate the robustness of image registration methods. When the RE value of the registered image pair is larger than the unregistered image pair, the registration work is effective. Otherwise, the registration work is considered a failure.

(20) $$ERR=\left(\frac{1}{M}\sum_{i=1}^{M}\sigma (RE_{i})\right)\times 100\%$$

(21) $$\sigma \left(RE_{i}\right)=\left\{\begin{array}{c} 1,\;\;\;\;\;RE_{i}>{\bar{RE}}_{i}\\ 0,\;\;\;\;\;others \end{array}\right.$$

where *M* is the number of test images, $RE_{i}$ denotes the *RE* value of the *i*th registered image pair, and $\bar{RE_{i}}$ denotes the *RE* value of the corresponding unregistered image pair.

## 3. Experiments and Results

The proposed method was tested on the LITIV dataset. The LITIV dataset was divided into single person, two people, and three people scenarios, and we selected 100 pairs of images from each of the three scenes. In this section, the superiority of the proposed method was proved from two aspects: (1) results of feature point extraction and matching and (2) results of image registration.

### 3.1. Experimental Results of Feature Point Extraction and Matching

**Figure 6:**
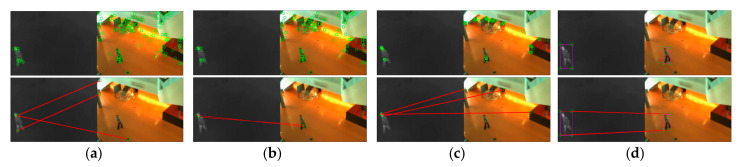
Experimental results of feature point extraction and matching in the single person scenario: (**a**) SIFT, (**b**) PSO-SIFT, (**c**) OS-SIFT, and (**d**) our method.

**Figure 7:**
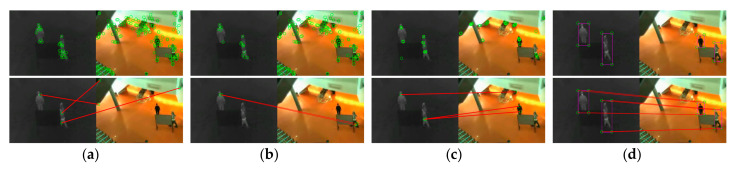
Experimental results of feature point extraction and matching in the two people scenario: (**a**) SIFT, (**b**) PSO-SIFT, (**c**) OS-SIFT, and (**d**) our method.

**Figure 8:**
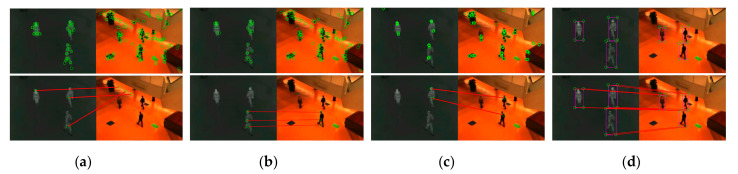
Experimental results of feature point extraction and matching in the three people scenario: (**a**) SIFT, (**b**) PSO-SIFT, (**c**) OS-SIFT, and (**d**) our method.

As exhibited in Figure 6, Figure 7 and Figure 8, we compared the proposed method with SIFT, PSO-SIFT, and OS-SIFT methods in terms of feature point extraction and matching [38,49,50]. SIFT is a classical method to remove mismatched points using the FSC algorithm, and PSO-SIFT and OS-SIFT are two advanced methods improved based on SIFT.

In terms of the feature point extraction, the SIFT, PSO-SIFT, and OS-SIFT methods can all extract lots of feature points. However, a large number of redundant points are produced in visible images, which brings much misguided feature information for the matching operation. It should be noted that the proposed method acquires an equal number of constraint points of infrared and visible images, which offers certain protection for accurate matching. In the aspects of feature point matching, it is difficult to get matched point pairs with the SIFT method. The PSO-SIFT method can obtain several correct matched point pairs, but sometimes these matched point pairs are too few to satisfy the requirement of affine transformation matrix solving. The OS-SIFT method rarely gets enough correct matched points pairs to finish infrared-visible image registration. In addition, these methods need to delete mismatched and superfluous point pairs, which increases the matching time. Unlike the SIFT, PSO-SIFT, and OS-SIFT methods, our method can exactly match all constrained points without eliminating redundant point pairs. Meanwhile, the proposed method can obtain enough matched point pairs to get a unique affine transformation matrix.

### 3.2. Experimental Results of Image Registration

In this section, we compare the registration results of the proposed method with five methods. SIFT, PSO-SIFT, and OS-SIFT are feature-based approaches. PSO-SIFT and OS-SIFT are modified based on SIFT. NTG and BlockNTG are intensity-based methods. BlockNTG is improved based on NTG [17,18]. The registration results of these methods are qualitatively and quantitatively analyzed as follows.

#### 3.2.1. Qualitative Analysis of the Registration Experiment Results

**Figure 9:**
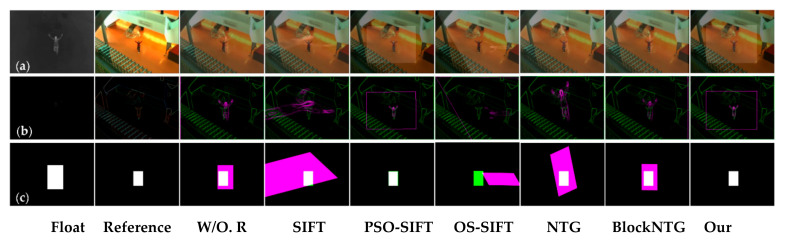
Registration results of different methods in the single person scenario: (**a**) the image pairs, (**b**) the gradient image pairs, and (**c**) the evaluation box pairs.

**Figure 10:**
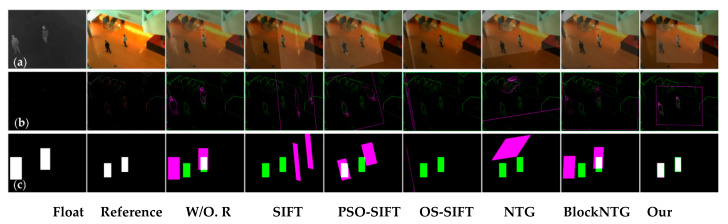
Registration results of different methods in the two people scenario: (**a**) the image pairs, (**b**) the gradient image pairs, and (**c**) the evaluation box pairs.

**Figure 11:**
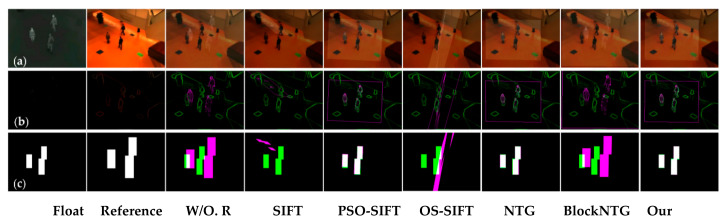
Registration results of different methods in the three people scenario: (**a**) The image pairs, (**b**) the gradient image pairs, and (**c**) the evaluation box pairs.

As shown in Figure 9, Figure 10 and Figure 11, the proposed framework was compared with five registration algorithms in three different scenarios. Float and reference images were infrared and visible images, respectively. The images in the W/O. R columns represent images without registration. The images in the (a), (b), and (c) rows qualitatively express the registration accuracy. The image pairs in the (a) rows represent the degree of alignment between the registered infrared image and the visible image. Gradient image pairs in the (b) rows give the accuracy of edge alignment between the registered infrared image and the visible image. Evaluation box pairs in the (c) rows reflect the degree of overlap between the registered infrared and visible images. The carmine and green lines denote the gradient of the infrared and visible images, respectively. The carmine, green, and white areas express pixels of boxes of the registered infrared image, visible image, and the overlap pixels area, respectively. As observed from Figure 9, Figure 10 and Figure 11, our method achieved better image alignment, edge alignment, and greater box overlap in these scenarios compared to other methods. The SIFT method was ineffective in all scenarios. The PSO-SIFT method sometimes obtained good registration. The OS-SIFT method was unable to register infrared-visible images well. The NTG method only achieved satisfactory registration in the three people scenario. The BlockNTG method did not get accurate registration in these scenarios. However, the qualitative analysis could not evaluate the proposed method specifically, so we conduct a quantitative evaluation next.

#### 3.2.2. Quantitative Analysis of Experimental Results

As shown in Table 2, Table 3 and Table 4, in order to validate the robustness, accuracy, and speed, the ERR, RE average value, RMSE average value, and average running time (Time) were introduced to evaluate the registration results of different methods. The values of RE and RMSE maintained the same trend in each scene, which illustrates that the proposed evaluation method is valid. In these scenarios, the ERR of our method was much higher than that of the other methods and reached 100%. It is obvious that the proposed method was robust to the variation of the environment. The RE and RMSE values of our method were the minimum among these methods under the different scenarios. Moreover, the RE and RMSE values were close to zero in the single person scenario, which demonstrates that our method can achieve almost completely accurate registration when images contain less information. The RE value of our method under the other two scenarios was also quite small, which indicates that our method can still achieve high precision registration when the information of images is relatively complex. The Time value acquired from our method was the least among these methods under the different scenarios, which expresses that our method has a fast calculation speed. The Time values of our method were 0.0736 s, 0.0727 s, and 0.0725 s, respectively. In other words, our method can register about 14 pairs of images per second. The experimental results convincingly prove that the proposed method obviously outperforms better performance than the methods mentioned in this paper.

**Figure 12:**
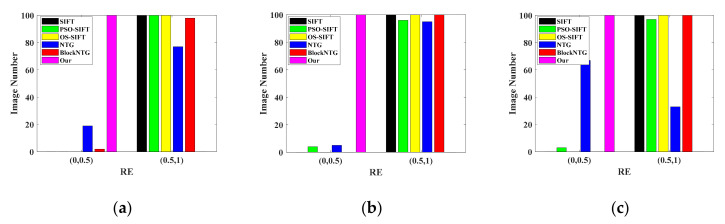
The statistic bar graphs of RE values under different scenarios: (**a**) the single person scenario, (**b**) the two people scenario, and (**c**) the three people scenario.

Figure 12 provides the statistic bar graphs of RE values under different scenarios. The RE value was divided into the ranges of (0,0.5) and (0.5,1). The RE value in (0,0.5) and (0.5,1) represented fine and poor registration results, respectively. As expressed in Figure 12, the registration results of SIFT and OS-SIFT methods were unsatisfactory in different scenarios. PSO-SIFT could realize excellent registration sporadically. The number of fine registration images of the NTG method in the three people scenario was greater than that of the single person and the two people scenarios, which indicates that the NTG method is more suitable for a scenario with rich information. The BlockNTG method obtained a few fine registration results in the single person scenario. Unlike the above five methods, the RE values of our method were all within the range of (0,0.5) in the three different scenarios, which shows the advantages of the proposed method in terms of robustness and accuracy.

## 4. Discussion

As described above, the experiments in this paper were conducted on the LITIV dataset. The experimental results and analysis of the feature point extraction, matching, and the image registration are provided.

As to the feature point extraction and matching, the proposed method was superior to the SIFT, PSO-SIFT, and OS-SIFT methods. The advantages of our method are reflected in the following aspects: (1) The matching precision is enhanced by capturing same number and sparse constraint points from object bounding boxes, (2) enough matched points are extracted to ensure the unique affine transformation matrix, and (3) the constrained points are exactly matched without removing redundant points, and the computing time is reduced.

As to the image registration, our method surpassed other methods mentioned in this paper in terms of robustness, accuracy, and speed. The SIFT and OS-SIFT methods could not achieve satisfactory image registration in different scenarios. The PSO-SIFT method could only successfully register a few infrared-visible images. The NTG method was most appropriate for the three people scenario. The BlockNTG method only obtained a few fine registration results in the single person scenario. The ERR values acquired by our method were all the largest among these methods, which means that our method has strong robustness to the variation of environment. Registered image pairs obtained by the proposed framework had excellent image alignment, edge alignment, and box overlap. The RE and RMSE values obtained by our method were the minimum among these methods, which indicates that our method has high registration accuracy. The proposed method improves registration accuracy from two aspects. On the one hand, constrained points are obtained by using the object detection algorithm, which introduces senior semantic information to ensure the accuracy of image registration. On the other hand, the proposed LV-rule method matches constrained points strictly one to one. The Time value of our method was the minimum among these methods under different scenarios, which illustrates that our method has a fast registration speed. There are two key points to speeding up registration. First, our method does not locate the feature points directly, but rather achieves region-level positioning by obtaining the object bounding box, and thus the speed of feature points extraction is increased. Second, the LV-rule method of matching points without subsequent mismatched elimination is proposed, and the complexity of the proposed method is reduced.

In summary, the proposed method shows better performance than the SIFT, PSO-SIFT, OS-SIFT, NTG, and BlockNTG methods in terms of registration accuracy, speed, and robustness.

## 5. Conclusions

An infrared-visible image registration framework based on the constrained point feature is presented in this paper. An object detection method was employed to obtain the constrained points, the LV-rule was designed to strictly and exactly match points, and an intelligent method was explored to evaluate registration accuracy and robustness. The proposed method was tested on the LITIV dataset and compared to the classic and state-of-the-art registration algorithms. Experimental results showed that the proposed method has high registration accuracy, speed, and good robustness to the variation of environment. Furthermore, the registration idea of this paper can be introduced to other image registration fields. Accurate and fast image registration lays a foundation for image fusion, target tracking, object recognition, and other tasks.

## Figures and Tables

**Table 1:** The comparison of the MA and time values of SSD, Faster R-CNN, and YOLOv3. The bold indicates the best value, ↑ denotes larger is better, and ↓ represents smaller is better.

**Method**	**MA (IR)↑**	**MA(VIS)↑**	**Time (IR)↓**	**Time (VIS)↓**
SSD	99.11%	98.33%	0.1170 s	0.1236 s
Faster R-CNN	**99.83%**	99.17%	0.3369 s	0.3523 s
YOLOv3	**99.83%**	**99.67%**	**0.0362 s**	**0.0363 s**

**Table 2:** The comparison of registration results under the single person scenario. The bold indicates the best value, ↑ denotes larger is better, and ↓ represents smaller is better.

Method	ERR↑	RE↓	RMSE↓	Time (s)↓
SIFT	7%	0.9701	744.4887	1.9167
PSO-SIFT	10%	0.5660	39.2626	4.6941
OS-SIFT	0%	*	*	1.4024
NTG	35%	0.5393	29.8977	18.3363
BlockNTG	39%	0.6922	51.8841	0.1583
Ours	**100%**	**0.0000**	**5.12E-14**	**0.0736**

*: The registration works of the method under this scene fail. ($RE_{i}\leq {\bar{RE}}_{i}$). $RE_{i}$ and ${\bar{RE}}_{i}$ represent the RE values of the *i*th registered image pair and unregistered image pair, respectively. The symbols ‘*’ in Table 2 mean the same thing. The value 5.12E-14 equals 5.12 × 10^−14^.

**Table 3:** The comparison of registration results under the two people scenario. The bold indicates the best value, ↑ denotes larger is better, and ↓ represents smaller is better.

Method	ERR↑	RE↓	RMSE↓	Time (s)↓
SIFT	2%	0.9190	769.1782	1.9570
PSO-SIFT	9%	0.5625	46.1094	4.5280
OS-SIFT	2%	0.9223	917.1556	1.6875
NTG	53%	0.7677	52.4014	18.7133
BlockNTG	9%	0.8668	110.3254	0.1713
Ours	**100%**	**0.0729**	**1.8655**	**0.0727**

**Table 4:** The comparison of registration results under the three people scenario. The bold indicates the best value, ↑ denotes larger is better, and ↓ represents smaller is better.

Method	ERR↑	RE↓	RMSE↓	Time (s)↓
SIFT	6%	0.9022	436.5665	1.9554
PSO-SIFT	6%	0.4674	29.5643	4.7568
OS-SIFT	3%	0.8886	210.6097	1.8521
NTG	82%	0.2854	17.9115	19.1369
BlockNTG	12%	0.9049	448.5638	0.1585
Ours	**100%**	**0.0819**	**3.0621**	**0.0725**

## Data Availability

Data sharing not applicable.
